# Mucormycosis in a surgical defect masquerading as osteomyelitis: a case report and review of literature

**DOI:** 10.11604/pamj.2016.23.16.8394

**Published:** 2016-01-26

**Authors:** Ashwini Kumar Mengji, Uday Shankar Yaga, Nishanth Gollamudi, Bhanu Prakash, Edunuri Rajashekar

**Affiliations:** 1 Department of Oral Medicine and Radiology, MNR Dental College and Hospital, Sangareddy, Telangana

**Keywords:** Diabetes mellitus hyphae, myiasis, PAS stain, zygomycosis

## Abstract

Mucormycosis is a rare, highly lethal opportunistic fungal disease affecting immune compromised and diabetic patients. Mucormycosis is considered as the 3^rd^ most common invasive mycosis after candidiasis and aspergillosis in debilitating patients. It is caused by the filamentous fungi of the class zygomycetes. The infection usually begins in the nose due to inhalation of fungal spores. This fatal fungal disease needs a prompt and early definitive diagnosis, aggressive surgical therapy and high dose anti-fungal therapy. Here, we present a case report of Mucormycosis in a 64 year elderly diabetic male patient who was previously operated for myiasis and also the extensive review of the literature of the mucormycosis.

## Introduction

Mucormycosis, an emerging angio invasive fungal infection, is considered as one of the most rapidly progressing and lethal form, caused by ubiquitous filamentous fungi mucorales in humans which usually begins in the nose and para nasal sinuses [1]. It is an opportunistic infection that occurs in patients with immune compromised, debilitating individuals or patients suffering from diabetes mellitus [2, 3]. Infection may be due to inhalation, ingestion or contamination of traumatized mucosa like ulcer or extraction socket by fungal spores [4]. It can be found in fruits, soil, dust, and manure and can be cultured from the nasal mucosa of normal persons, where it may not cause clinical signs of infection [5]. The organisms are aerobic, but can live two to five days in vitro. Although infection usually occurs after inhalation through the nose or mouth, a skin laceration can also become an opening for mycotic entry [6].

## Patient and observation

**Figure 1:**
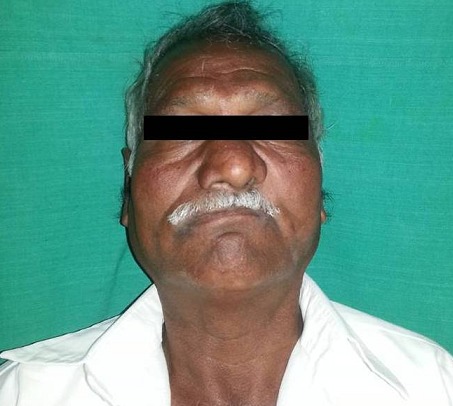
Extra oral image showing sunken appearance of face and loss of naso labial fold on left side

**Figure 2:**
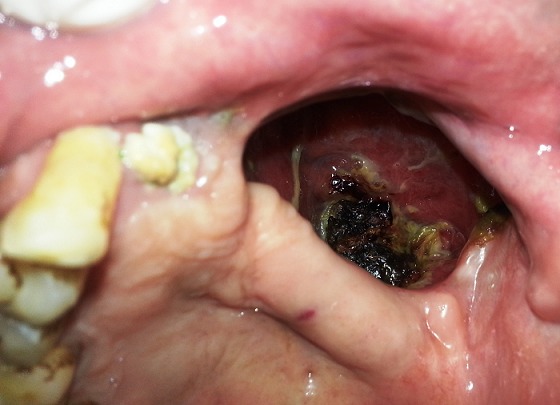
Intra oral image showing surgical defect with necrotic bone and necrotic slough

A 64 year old male patient, farmer by profession, reported to outpatient department of Oral Medicine, Diagnosis and Radiology with a chief complaint of pain and pus discharge in relation to the left upper back tooth region since 4 months. Past history revealed that patient was asymptomatic 4 months ago, later he developed pain and pus discharge which was slow in onset, localized, dull in nature, intermittent with no aggravating or relieving factors. Further dialog history revealed that patient also experienced numbness on left side of the face since 2 months. Past medical history revealed that patient was hypertensive and diabetic since 5 and 4 years respectively and currently on medication. Past dental history and supportive investigations revealed that patient underwent check up 4 years ago at a ENT doctor where it was provisionally diagnosed as a case of Nasal myiasis, surgical debridement of the lesion was done and maggots were retrieved. Plain and contrast computed tomography of brain revealed a sharply defined, mildly hyperdense space occuping lesion of 24 x 19 mm in left temporal region and effaced sulcal spaces which showed mild enhancement on contrast with mild hyperostosis with widening of diploic spaces of greater wing of sphenoid bones which was suggestive of a meningioma. Following which patient was admitted at a Private dental hospital, treatment protocol was informed to the patient and after obtaining informed consent from the patient, he was posted for surgery under general anesthesia. Maxillectomy on the left side was done, after 2 months of regular debridements and obtaining sterile cultures from the surgical wound, acrylic palatal obturator was fabricated making alginate impressions. Extra oral examination revealed a gross facial asymmetry with sunken appearance with loss of normal nasolabial fold on the left side of the face (Figure 1). TMJ and lymph nodes examination showed no abnormality. Intra oral examination showed a surgical defect in relation to the left maxillary quadrant with well defined borders. The depth of the lesion showed a grayish black necrotic pseudo membranous slough with yellowish areas interspersed all over (Figure 2). Other intra oral findings were multiple missing teeth in left and right maxilla and periodontally compromised mandibular teeth. Based on the past history and intra oral examination, a provisional diagnosis of osteomyelitis of the surgical defect was given. Other lesions such as Tuberculosis, Squamous cell carcinoma, Tertiary syphilis, fungal infections such as Aspergillosis and Mucormycosis were considered.

**Figure 3:**
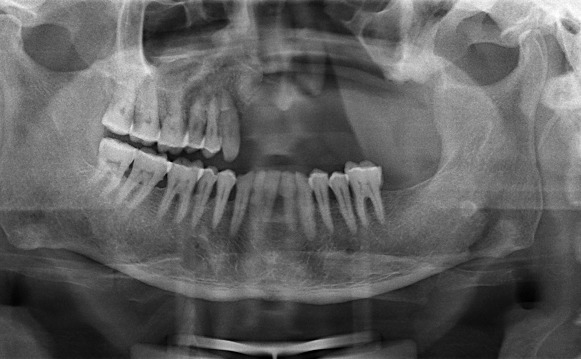
Panaromic radiograph showing multiple missing teeth and features suggestive of left maxillectomy

**Figure 4:**
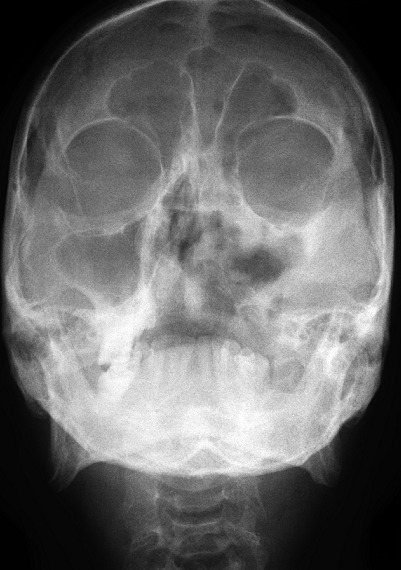
Para nasal sinus view showing irregular diffuse radiolucency involving left maxillary sinus suggestive of osteomyelitis

**Figure 5:**
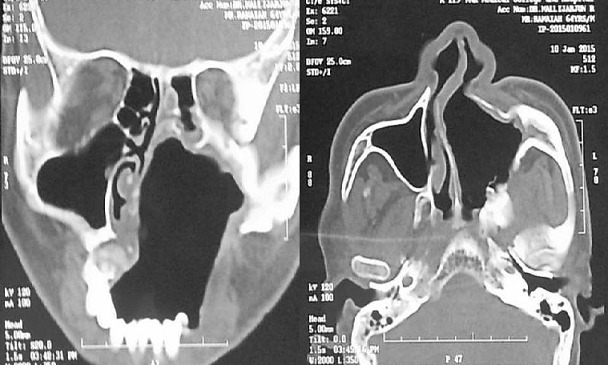
Computed tomography image sagittal and coronal section showing irregular radiolucency suggestive of post operative left maxillectomy, deviated nasal spine to right, left ethmoidal and sphenoidal sinusitis, Changes seen involving left pterygoid plates and posterior wall of maxillary antrum suggestive of residual changes of Osteomyelitis

**Figure 6:**
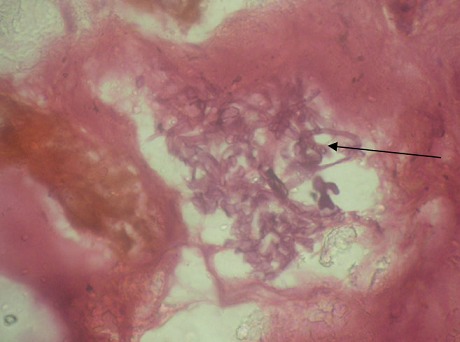
PAS stained section under low power showed cellular connective tissue stroma with large non septate fungal hyphae (in black arrow) along with few areas of necrosis

**Figure 7:**
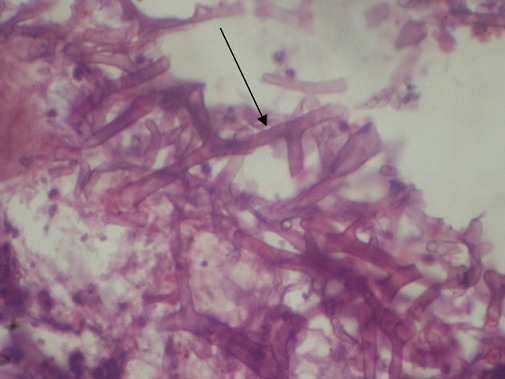
Hematoxylin and eosin stained histopathological section showed cellular connective tissue stroma with large non septate fungal hyphae along with few areas of necrosis. focal areas of mixed inflammatory cell infiltrate were evident

Radiologic investigations of Panaromic radiograph and computed tomography were advised. Panaromic radiograph revealed multiple missing teeth (12 11 21 22 23 24 25 26 27 37) and huge surgical defect in favor of left maxillectomy (Figure 3). Paranasal sinus radiograph view showed radiolucency which is diffuse, with irregular borders involving the left maxillary antrum and deviated nasal spine and changes in favor of left maxillectomy (Figure 4). Computed axial tomography revealed in favor of post operative left maxillectomy, deviated nasal spine to right, left ethmoidal and sphenoidal sinusitis. Changes seen involving left pterygoid plates and posterior wall of maxillary antrum suggestive of residual changes of Osteomyelitis (Figure 5). Biochemical investigations such as complete blood picture, serum creatinine were within normal limits with increased Erythrocyte sedimentation rate and Glycoslyated hemoglobin was 9.2% suggestive of diabetes mellitus. Incisional biopsy was done and the specimen was obtained from the left maxillary antrum through endoscope under local anesthesia and was sent to Culture sensitivity tests which showed positivity for bacterial and fungal growth on KOH culture media. The specimen was further sent for Periodic acid Schiff staining which showed large non septate fungal hyphae branched at right angles (Figure 6). The specimen was sent for histopathological examination (Figure 7) which showed cellular connective tissue stroma composed of mixed inflammatory cell infiltrate predominantly neutrophils and lymphocytes, numerous large non septate fungal hyphae branching at right angle along with few areas of necrosis. There is presence of ciliated columnar epithelium along with few bony trabaculae and extensive areas of hemorrhage along with super added bacterial infection suggestive of “mucormycosis. Correlating clinically, radiologically and histologically a final diagnosis of **CHRONIC OSTEOMYEILITIS WITH MUCORMYCOSIS** was given. Our patient underwent extensive unilateral endoscopic nasosinusal surgery with debridement of left maxillary sinus, anterior and posterior ethmoidal cells and extensive removal of invaded mucosa under general anesthesia. Extensive fungal masses and necrotic slough were excised from left maxillary and sphenoidal sinus. Necrotic mucosa was removed till the healthy margins were visible which showed initial signs of bleeding. Liposomal Amphotericin B (5 mg/kg) was started immediately after the surgery and dosage was increased to (7.5 mg/kg) after 3 weeks. A week later patient underwent hyperbaric oxygen therapy for 21 dives, each session lasting for 150 min along with systemic delivery of antifungal drugs orally for time period of 4 weeks. Post operative visit of the patient showed negative cultures with no morbidity and patient was delivered an acrylic obturator with soft liners.

## Discussion

Mucormycosis is a rare fungal infection seen in the immuno compromised previously referred to as zygomycosis [7]. This condition was first described by Paltauf in 1885 in human beings [8]. Though this fungus is ubiquitous in the environment (damp places such as soil, composting vegetation, and bread etc), our body is protected by the immune system and is therefore rare. Risk factors for the disease include diabetes mellitus, leukemia, blood dyscracias, immunesuppressive conditions such as Graft versus host diseases etc [9]. It may also manifest in immu¬no competent persons [10]. Its occurrence in diabetic patients can be attributed to that fact that low ph or the acidosis, hyperglycemic state. In these patients the phagocytic capacity of granulocytes is reduced, increasing the free ferric ions availability which favors growth of the fungus and enzyme ketoreductase of Rhizopus fungi utilizes the available ketone bodies. Mucorales are ferrophilic fungi. Acidosis reduces the binding of iron to transferrin; in turn, available free iron helps in proliferation of the Mucorales [11]. Mucormycosis generally progresses in three stages [9]. The first stage occurs after the inhalation of the fungal spores and invasion of the para¬nasal sinuses, resulting necrosis of the nasal mucosa, turbinates, or hard palate. The second stage is characterized by direct extension of the disease into the maxillary sinus. During the last stage, the infection spreads into the cribriform plate or the orbit [9].

### Clinical presentation

Mucormycosis has various clinical presentations. Six variants have been described by Eisenberg et al namely rhinocerebral, pulmonary, gastrointestinal, cutaneous, nervous system and disseminated. among these, rhinocerebral is the most common type accounting for one -third to one half of the reported cases [12], which is further categorized into two forms, highly fatal form called the Rhino-Orbital variant affecting ophthalmic and internal carotid arteries and a less fatal and more common variant Rhino-Maxillary affecting spheno palatine and greater palatine arteries [13]. The Rhino cerebral variant is the most common form of mucormycosis in patients with diabetic ketoacidosis accounting for 70% of published cases [14]. Maxillary, frontal, ethmoidal and sphenoidal sinuses are the most frequent location and the mean age of patients was 38.8 years. This ubiquitous fungus is angioinvasive resulting in thrombosis and ischemic tissue necrosis. A black necrotic eschar is the most characteristic and the pathognomic lesion. Patients usually present with malaise, headache, facial pain, swelling and low grade fever [6]. Necrosis of the maxilla is usually rare due to its rich vascularity. But the angioinvasive fungus which causes thrombosis and necrosis may advances to the palate leading to avascular necrosis. The maxillary sequestration and necrosis is a evidence to the necrotizing and invasing potential of the fungus. If untreated or unnoticed the disease has the capacity to spread throughout the entire face, resulting in necrosis of maxilla, orbit penetrating deeper into cranium causing mortality [15]. Initial symptoms are usually those of a sinusitis or orbitary cellulitis. The most frequent intracranial complications are epidural and subdural abscesses and cavernous and longitudinal sinus thrombosis [16]. Symptoms of sinusitis or periorbital cellulitis, low-grade fever, facial swelling & numbness, blurred or decreased vision, soft tissue swelling, palatal ulcer or perforation of the palate, are the most common. Other symptoms include epiphora and ophthalmoparesis, nasal stuffiness and epistaxis [17, 18]. It originates usually either from the nose (inhalation) or from contamination of the wound directly and subsequent dissemination to other parts. When it arises intra orally from the nose and para nasal sinuses, usually causes ulceration and perforation of the palate leading to necrosis, which appears as necrotic pseudomembranous slough. Cavernous sinus thrombosis is a serious and fatal complication of maxillary infections. If the disease invades the mouth, a black necrotic eschar is usually found on the palate [14]. Asexual spores are responsible for infection in humans. These air borne spores reach oral or nasal mucosa. In healthy individuals growth or reproduction of these spores are usually resisted by phagocytes, however in immune compromised states or individuals where the host response is impaired, infection progresses. These hyphae have affinity to blood vessels, reach them, breach the lumen and propagate within the vessel walls leading to cascade of events such a thrombosis, ischemia, infarction, necrosis, gangrene and finally sequestration of the affected tissue [14].

### Radiology

Plain film radiography is nonspecific and showing haziness in the antrum unilaterally. The gold standard imaging method is contrast enhanced computed tomography which shows demonstrated opacification without fluid levels with nodular thickening of the sinus and spotty destruction of the walls of paranasal sinuses [15, 19]. Computed tomography finding are usually non specific or non characteristic. Mc Donogh hypotheticated that any diabetic patient in a ketoacidotic state presenting with clinical and radiographic features of sinusitis should be suspected as having mucormycosis until proven otherwise. Black turbinate sign appears as non enhancing lesion in early stages of diseases is characteristic of the infected necrotic tissue on Magnetic resonance imaging (MRI) [20]. MRI is useful in determination of extension of the disease and pre-surgical planning. T2-weighted MRI images shows cerebral infection and contrast enhanced radiography may detect early vascular invasion [19]. Mostly patients with early case of mucormycosis may show normal CT and MRI study. Fungus is identified by hematoxylin and eosin stain, cultural sensitivity of the specimen and can confirm by Grocott's silver methenamine special staining technique which show the organism in vessel walls [21]. However, a polymerase chain reaction technique helps in identifying specific pathogenic strain and further treatment protocol.

### Histopathological picture

Histopathological picture shows the characteristic ribbon like branching, smaller width non septate hyphae which are prominent and long, acute angled [17]. Hyphae are better visualized with PAS or silver strains. As the fungus is angio invasive it is commonly found in close proximity with the necrotic vessel walls. Usually tissue shows non-specific inflammatory cell infiltrate, with necrosis and granulation tissue along with the hyphae.

### Management

Early diagnosis, prompt management (aggressive surgical intervention, concurrent anti fungal therapy and supportive measures such as hyperbaric oxygen therapy) is critical because of the invasive and fulminate course of the disease and has decreased the mortality rates drastically from 84% to 40 in a span of 40 years [19]. Our patient underwent extensive surgical debridement under general anesthesia, with concurrent antifungal treatment and hyperbaric oxygen therapy for 21 dives. This angio invasive fungal infection needs extensive surgical debridement along with the healthy margins and immediate anti fungal treatment especially potent drugs like Amphotericin B (AmB) in the dose of 5 mg / kg body weight through intra venous route. Other lines of treatments such as reversal of predisposing factors such as diabetes mellitus should go simultaneously [22, 23]. AmB is a natural antibiotic belonging to the polyene group which acts by binding the hydrophilic part of the drug to the ergosterol present in the cell membrane of the fungus forming transmembrane channels causing depolarization of the membrane, increasing the membrane permeability to protons. This causes the leakage of intracellular contents leading to cell lysis. It also has affinity to lipids present in the human cell membrane but to lesser extent than the ergosterol. It binds to the cholesterol present in the human cell membranes contributing to the toxic effects of the drug such as nausea, vomiting, rigors, fever, hypertension/hypotension, hypoxia etc [24, 25]. AmB is drug of choice and considered as the best form of treatment in such disseminated, life-threatening fungal infections, which showed survival rate of up to 72% [26]. When treating a patient with AmB few points such as monitoring of electrolytes including magnesium and phosphates and renal function tests as it is known to have few nephrotoxic effects [24]. Although AmB remains as main drug of choice in such lesions, invitro studies showed that, Posaconazole, an extended spectrum traizole, demonstrated anti fungal activity for serious invasive cases, against the Zygomyces species.

### Role of hyperbaric oxygen therap

Hyperbaric oxygen shows anti fungal activity by inhibiting lactic acidosis, enhanced phagocytosis and increased activity of polymorphonuclear leukocytes. It also contributes healing by increasing the oxygen tension to the hypoxic areas [27, 28]. When the infection spreads intracranially and shows poor response to conventional treatments Iron-chelating agents like Deferasirox can be administered although with varying results [22]. After obtaining negative cultures from the lesion, usually the surgical defect is closed with an obturator to prevent nasal regurgitation and improvement of aesthetics.

## Conclusion

Mucormycosis is considered as aggressive, uncommon, fulminating, fatal, invasive, fungal infection with poor prognosis. Diabetes mellitus is the most common pre disposing factor for the fungal infection due to poor defense although other immunocompromised states do contribute to the pathogenesis of this disease. It manifests as many forms but Rhino cerebral type is the most common variant affecting paranasal sinuses. Early diagnosis and prompt management is very crucial because it is fatal when it involves multiple systems. AmB is usually drug of choice, Hyperbaric oxygen therapy may act as an adjuvant in promoting wound healing and increasing phagocytosis. Stomatologists play an important role in early diagnosis thus reducing the mortality and morbidity associated with the disease. Interdisciplinary approach with Dental specialists such as Oral and Faciomaxillary surgeons, Prosthodontists, ENT surgeons, ophthalmologists and neurologist play an important role in the management of this debilitating disease.
